# Evaluation of the Diagnostic Accuracy of the T2Resistance Panel (Research Use Only) in Patients With Possible Bacterial Bloodstream Infections

**DOI:** 10.31486/toj.24.0101

**Published:** 2025

**Authors:** Deborah Ashcraft, Jesse St Pierre, Heather Davis, Susan Scariano, Maria Latsis, Royanne Vortisch, Samantha Smith, George Pankey

**Affiliations:** ^1^ Infectious Disease Translational Research, Ochsner Clinic Foundation, New Orleans, LA; ^2^ The University of Queensland Medical School, Ochsner Clinical School, New Orleans, LA; ^3^ Clinical Research, Ochsner Clinic Foundation, New Orleans, LA

**Keywords:** *Drug resistance–microbial*, *genes–MDR*, *rapid diagnostic tests*

## Abstract

**Background:**

Early identification and antimicrobial susceptibility testing (AST) of bloodstream pathogens are important for promptly determining the appropriate therapy. Currently, positive blood culture results (identification and AST) are reported in 2 to 4 days. The T2Resistance (T2R) Panel (T2 Biosystems, Inc) uses DNA amplification with magnetic resonance from 3 mL of whole blood for direct detection of 13 antibiotic resistance genes: *bla*_KPC_, *bla*_NDM_, *bla*_VIM_, *bla*_IMP_, *bla*_OXA-48_, *bla*_CTX-M-14/15_, AmpC *bla*_CMY/DHA_, *van*A/B, and *mec*A/C. We compared the accuracy of T2R testing to AST for positive blood cultures.

**Methods:**

This investigator-sponsored, single-center study prospectively enrolled 802 patients with a standard of care blood culture. Five hundred forty-seven patients had adequate blood for culture and T2R testing. Blood cultures with positive isolates were identified, and AST was performed. Blood samples with positive blood cultures were tested with the T2R Panel.

**Results:**

Blood cultures were positive for 58/547 (10.6%) patients. Contaminants (18/547 [3.3%]) were excluded. T2R testing results (n=31) showed 2 *bla*_CTX-M-14/15_ genes with 100% sensitivity; the remaining gram-negative resistance genes were not detected, so sensitivity could not accurately be determined. Specificity was 100% for the 16 gram-negative bacilli. Three enterococci and 2 *Staphylococcus aureus* showed 100% sensitivity/specificity. However, 10 coagulase-negative staphylococci showed 17% sensitivity/100% specificity. Antibiotic resistance genes identified were 2 *bla*_CTX-M-14/15_, 2 *mec*A/C, and 1 *van*A/B. T2R testing results were obtained in an average of 7 hours.

**Conclusion:**

T2R testing is highly specific (100%) for the 13 antibiotic resistance genes on the panel. Sensitivity was 100% for the genes detected but was low (17%) for coagulase-negative staphylococci. T2R testing has the potential to diagnose certain antibiotic resistance genes from bacterial bloodstream infections in hours vs the days required for a positive blood culture with AST. Additional studies that include larger numbers of samples with antimicrobial resistance genes are needed.

## INTRODUCTION

In 2020, the World Health Organization released the *Global Report on the Epidemiology and Burden of Sepsis: Current Evidence, Identifying Gaps and Future Directions* with the aim of improving the prevention, diagnosis, and clinical management of sepsis.^1^ According to the report, there were approximately 49 million cases of sepsis and 11 million sepsis-related deaths worldwide in 2017, accounting for approximately 20% of all deaths globally.^1^

Blood culture is the primary method for diagnosing bloodstream infections. Currently, the gold standard for diagnosing a bloodstream infection is a positive blood culture with the results—identification and antimicrobial susceptibility testing (AST)—reported in 2 to 4 days. Blood cultures are often positive within 24 hours, and a Gram stain is performed and reported. The blood culture broth is then subcultured to an agar plate and incubated for an additional 18 to 24 hours. Once growth on the plate is visible, identification and AST can be performed, which may take an additional 24 hours.

Early identification and phenotypic AST of bloodstream pathogens are important for prompt appropriate therapy. However, in addition to poor turnaround time, blood cultures have a low sensitivity (approximately 70%) in critically ill patients.^2-5^ Prior administration of antibiotics may further decrease the sensitivity of a blood culture. Better diagnostic tests are needed for sepsis management. Genotypic (molecular-based) diagnostic tests, which can detect organisms and significant antimicrobial resistance genes from blood culture broth once the blood culture is positive, are now available. The presence or absence of antimicrobial resistance gene markers can be used to predict phenotypic AST results to help guide physicians to rapid appropriate therapy.^6^

T2 Biosystems, Inc developed a magnetic resonance-based molecular diagnostic device (T2Dx) that uses US Food and Drug Administration (FDA)-cleared panels to identify the most clinically significant bacteria (T2Bacteria Panel) and fungi (T2Candida Panel) directly from a 3 to 4 mL whole blood sample. This sample is collected simultaneously with the blood culture sample. Unlike a blood culture, which is loaded onto an instrument and incubated until positive (usually 24 hours), T2 testing can be performed immediately, with results available in 3 to 5 hours when the blood sample is loaded in the T2Dx Instrument immediately after collection. Intact pathogen cells are concentrated in the whole blood sample and lysed to release target DNA. After amplification, the target amplicon is hybridized with superparamagnetic particles that are detected by T2 magnetic resonance. The T2Bacteria Panel detects the following pathogens: *Enterococcus faecium, Staphylococcus aureus, Klebsiella pneumoniae, Pseudomonas aeruginosa, Escherichia coli, and Acinetobacter baumannii* (the latter is approved in Europe only). The T2Candida Panel detects common *Candida* pathogens that account for >90% of candidemia at most hospitals: *Candida albicans/Candida tropicalis, Candida glabrata/Candida krusei*, and *Candida parapsilosis*. Published studies reported a mean sensitivity of 93.7% for both the T2Bacteria and T2Candida panels and mean specificity of 97.1% and 97.2%, respectively.^7-14^ In addition, Giannella et al performed a meta-analysis of 14 primary studies comparing the T2Bacteria and T2Candida panels to blood culture.^15^ Patients with a positive T2 test vs blood culture received targeted antimicrobial therapy faster (–42 hours; *P*<0.001) and had a shorter hospital stay (–4.8 days; *P*=0.03). Patient mortality rates were comparable for T2 panels and blood culture.^15^

The T2Resistance (T2R) Panel (T2 Biosystems, Inc) (available for research use only in the United States, pending clearance by the FDA for use as a diagnostic test) uses the same technology for direct detection of 13 antibiotic resistance genes from any bacterial species, with sensitivity as low as ≤10 CFU/mL: (a) the major carbapenem resistance genes listed in the “2019 Antibiotic Resistance Threats Report” from the Centers for Disease Control and Prevention^16^ (*bla*_KPC_, *bla*_NDM_, *bla*_VIM_, *bla*_IMP_, and *bla*_OXA-48_); (b) the extended-spectrum beta-lactamases (*bla*_CTX-M-14_ and *bla*_CTX-M-15_); (c) the beta-lactamases active against ampicillin carbapenemase (AmpC *bla*_CMY_ and *bla*_DHA_); (d) the gram-positive *van*A and *van*B resistance genes that are responsible for vancomycin resistance in *Enterococcus*; and (e) the *mec*A and *mec*C resistance genes that are responsible for methicillin resistance in *Staphylococcus*. T2R testing results are available in 3 to 5 hours.^17^ Ideally, samples would be tested with both the T2Bacteria Panel and the T2R Panel simultaneously so that bacterial identification could be reported along with the presence or absence of resistance mechanisms.

Preliminary data from studies of the T2R Panel in Italy were reported in 2021 and 2022.^18,19^ For this study, we evaluated the diagnostic accuracy and turnaround time of the T2R Panel compared with the phenotypic AST for positive blood cultures collected from patients at a medical center in the southeastern United States.

## METHODS

### Patient Enrollment

We conducted an investigator-sponsored study at Ochsner Medical Center in New Orleans, Louisiana, to evaluate the diagnostic accuracy of the T2R Panel. A total of 802 patients were prospectively enrolled from January 8, 2021, to October 6, 2021. The inclusion criteria included all patients ≥18 years for whom a blood culture was ordered as part of the standard of care and who provided verbal informed consent to participate in the study. The exclusion criteria prevented enrollment of any patient who was previously enrolled in the study or did not have at least 3 K_2_EDTA tubes with ≥3 mL blood collected for T2R testing. An adequate volume of blood for culture and T2R testing was collected for 547 patients (68.2%): 368 patients from the emergency department, 173 patients from intensive care units, and 6 patients from hospital floors. The institutional review board approved the study protocol (#2020.009).

### Blood Collection and Sample Testing

Blood samples were collected for blood culture and T2R testing by filling 1 aerobic and 1 anaerobic blood culture bottle first, each with 5 to 10 mL of blood, followed by 3 K_2_EDTA vacutainer tubes (Becton, Dickinson and Company) with 3 to 4 mL blood per tube for T2R testing. The T2R samples were collected immediately following the collection of blood for culture from the same collection site with the same needle or catheter. All blood samples for T2R testing were stored frozen at –70 °C for an average time of 2 months until tested.

Blood cultures were performed according to hospital practices using the BD BACTEC FX instrument (Becton, Dickinson and Company). Cultures with no growth after 5 days were reported as negative. Species were identified from positive cultures (growth on agar media) using matrix-assisted laser desorption/ionization-time of flight mass spectrometry (MALDI-TOF MS) (Bruker Daltonics). The *time to species ID* (identification) was the time from the start of the blood culture incubation to the reporting of the species identification. The *time to MIC* (minimum inhibitory concentration) was the time from the start of the blood culture incubation to the reporting of the AST for the organism. All blood culture testing was performed in the hospital microbiology laboratory by laboratory staff.

For this study only, T2R testing was performed for all patients with a positive blood culture result, independent of AST result. In a clinical setting, the T2R sample would be collected simultaneously with a blood culture sample and tested immediately, with results reported in 3 to 5 hours. Blood cultures reported as a probable contaminant were not tested with T2R. The *time to T2R Panel result* was defined as the time from sample loading to the result reporting on the T2Dx Instrument (T2 Biosystems, Inc). T2R testing was performed using 2 T2Dx Instruments in the Infectious Disease Translational Research laboratory by laboratory staff. Up to 7 samples can be loaded into the T2Dx Instrument at once. The number of samples loaded affects the time to result. Because all blood samples for T2R testing were frozen for this study, 7 samples were usually loaded for each batch of testing, so the time to T2R Panel result may not be predictive of clinical use.

### Data Analysis

Sensitivity and specificity were calculated for each group of resistance genes: gram-negative bacilli (*bla*_KPC_, *bla*_NDM_, *bla*_VIM_, *bla*_IMP_, *bla*_OXA-48_, *bla*_CTX-M-14_, *bla*_CTX-M-15_, *bla*_CMY_, and *bla*_DHA_); enterococci (*van*A, *van*B); and staphylococci (*mec*A, *mec*C). The antibiotic susceptibility phenotype of the organism grown in blood culture was used as a standard to compare to the resistance genes detected in the T2R Panel: for enterococci, vancomycin resistance (*van*A, *van*B); for staphylococci, oxacillin resistance (*mec*A, *mec*C); for gram-negative bacilli, ceftriaxone resistance (*bla*_CTX-M-14_, *bla*_CTX-M-15_), meropenem or ertapenem resistance (*bla*_KPC_, *bla*_NDM_, *bla*_VIM_, *bla*_IMP_, *bla*_OXA-48_), or piperacillin-tazobactam, cefazolin, ampicillin-sulbactam, or ampicillin-clavulanate resistance (AmpC *bla*_CMY_ and *bla*_DHA_). Sensitivity was calculated as the number of true positives/true positives + false negatives, and specificity was calculated as the number of true negatives/true negatives + false positives.

## RESULTS

A total of 547 patients—59% male, 41% female, ages 19 to 98 years (mean age 61 years)—were included in the study. Positive blood cultures were reported in 58/547 (10.6%) patients: true positives for 40/547 (7.3%) patients (40 bacteria, 3 yeast) and probable contaminants for 18/547 (3.3%) patients. Contaminants were excluded from this study. Two blood cultures had multiple isolates: 1 grew *E faecium, Staphylococcus epidermidis*, and *E coli*, while another blood culture grew *S epidermidis, Staphylococcus haemolyticus*, and *Staphylococcus capitis*. In addition, 13 other patient samples were not evaluated: 5 for insufficient blood volume, 3 for invalid internal control, and 5 for AST not routinely performed (1 *Bacteroides*, 1 *Streptococcus*, and 3 *Candida*).

The Figure provides an overview of patient enrollment, blood cultures, and T2R testing results. Based on blood culture susceptibility testing, T2R testing produced 26 concordant results. Within the concordant results group, the T2R Panel was positive for 5 resistance genes: 2 *bla*_CTX-M-14/15_ (*K pneumoniae* and *E coli*), 2 *mec*A/C (1 methicillin-resistant *S aureus,* 1 methicillin-resistant *S epidermidis*), and 1 *van*A/B (*E faecium*) (Figure, Concordant T2R results).

**Figure.**
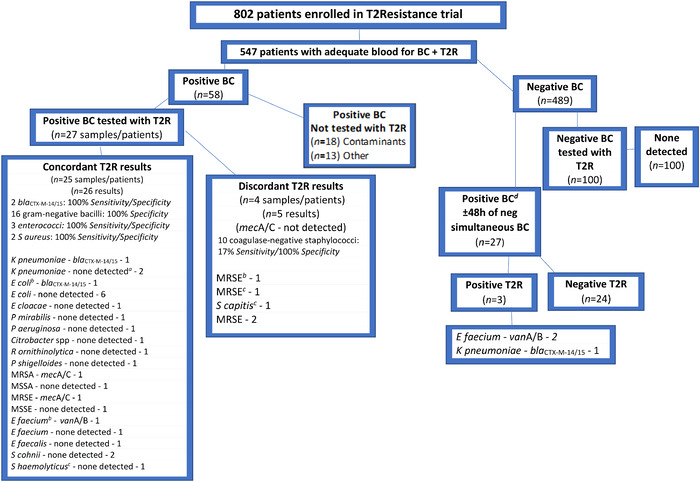
T2Resistance Panel results compared to simultaneously drawn blood culture results from patients with a possible bloodstream infection. ^a^None detected=no target genes were detected with T2R testing. ^b^Blood culture had 3 bacterial species: *Enterococcus faecium* (vancomycin resistant); *Escherichia coli* (ampicillin-sulbactam, amoxicillin-clavulanate, cefazolin, cefepime, and ceftriaxone resistant); and methicillin-resistant *Staphylococcus epidermidis* (MRSE). T2R Panel results were positive for *van*A/B and positive for *bla*_CTX-M-14/15_. ^c^Blood culture had 3 bacterial species: methicillin-resistant *Staphylococcus epidermidis, Staphylococcus haemolyticus* (oxacillin susceptible), and *Staphylococcus capitis* (oxacillin resistant). In the T2R Panel results, a *mec*A/C gene was not detected. ^d^Patients having a negative simultaneous blood culture but a different positive blood culture collected within 48 hours of the original simultaneous blood culture/T2R sample had their original negative simultaneous blood culture tested with the T2R Panel. BC, blood culture; *E cloacae, Enterobacter cloacae; E coli, Escherichia coli; E faecalis, Enterococcus faecalis; E faecium, Enterococcus faecium*; h, hour; *K pneumoniae, Klebsiella pneumoniae*; MRSA, methicillin-resistant *Staphylococcus aureus*; MRSE, methicillin-resistant *Staphylococcus epidermidis*; MSSA, methicillin-susceptible *Staphylococcus aureus*; MSSE, methicillin-susceptible *Staphylococcus epidermidis*; neg, negative; *P aeruginosa*, *Pseudomonas aeruginosa*; *P mirabilis, Proteus mirabilis; P shigelloides, Plesiomonas shigelloides; R ornithinolytica, Raoultella ornithinolytica; S aureus, Staphylococcus aureus; S capitis, Staphylococcus capitis; S cohnii, Staphylococcus cohnii; S haemolyticus; Staphylococcus haemolyticus*; T2R, T2Resistance Panel.

One patient had a positive blood culture with *E faecium* and methicillin-resistant *S epidermidis*. T2R testing was negative for *mec*A/C (did not correlate with phenotypic AST, oxacillin-resistant [Figure, Discordant T2R results]); positive for *van*A/B (correlated with phenotypic AST, vancomycin-resistant [Figure, Concordant T2R results]); and positive for *bla*_CTX-M-14/15_ (Figure, footnote b). Based on the absence of a strain that would typically carry *bla*_CTX-M-14/15_ in the blood culture, the detection of this resistance gene was thought to be discordant. After review of the patient's medical record, however, the *bla*_CTX-M-14/15_ result from T2R testing was believed to be associated with other evidence of infection and considered to be a true positive. Briefly, this patient was diagnosed with acute osteomyelitis in the right foot. Cultures from this abscess grew *E coli* 14 days prior to the simultaneous blood culture/T2R sample collection. Phenotypic AST reported all antimicrobials as susceptible. Prior to the T2R sample collection, the patient was administered several doses of antibiotics, including piperacillin-tazobactam, cefepime, and ceftriaxone. It is likely that the organism was circulating in the patient's bloodstream, but conventional blood culture was unable to detect it. The T2R Panel detected *bla*_CTX-M-14/15_, which is presumed to be valid because the patient was treated with antibiotics (including ceftriaxone) prior to the T2R sample collection, and a tracheal aspirate later grew an extended-spectrum beta-lactamase + *E coli* with phenotypic AST reported as resistant to ampicillin-sulbactam, amoxicillin-clavulanate, cefazolin, cefepime, and ceftriaxone (Figure, Concordant T2R results). The patient subsequently experienced a right-sided cerebral hemorrhage and died.

The T2R Panel was discordant with blood culture susceptibility in 4 patients, which were reported as 4 false-negative *mec*A/C gene results for methicillin-resistant *S epidermidis* and 1 false-negative *mec*A/C gene result for methicillin-resistant *S capitis* (Figure, Discordant T2R results).

### T2R Testing/Antimicrobial Susceptibility Testing Discordant Analysis

For 9 blood culture–positive isolates (from 8 patients) that had AST showing resistance or borderline susceptibility results but that tested negative with the T2R Panel, additional genetic testing of the bacterial isolate was performed. Briefly, overnight growth from the 9 bacterial isolates (4 methicillin-resistant *S epidermidis*, 1 *S capitis*, 2 *E coli*, 1 *P aeruginosa*, and 1 *Citrobacter* spp) was used to prepare bacterial cell pellets at a final concentration of 10^6^ cells/μL. Bacterial cells were lysed with multiple freeze/thaw cycles at –80 °C. Polymerase chain reaction reagents containing primers for the 13 antibiotic resistance genes were mixed with the lysate and amplified. The resulting amplicons were quantified and normalized to 20 ng/μL before being shipped for library preparation and next-generation sequencing (NGS). The sequencing data were processed and analyzed using a bioinformatics pipeline consisting of sequence trimming, joining, collapsing, and identification by the basic local alignment search tool (BLAST) against curated databases (Azenta, Inc).

The NGS analysis (Table 1) showed that the 5 isolates of coagulase-negative *Staphylococcus* (4 methicillin-resistant *S epidermidis* and 1 methicillin-resistant *S capitis*) harbored the *mec*A/C gene. These T2R results were classified as false negatives and are included in the Figure in the Discordant T2R results block. None of the antibiotic resistance genes on the T2R Panel was detected by NGS in the 4 gram-negative bacilli (2 *E coli,* 1 *P aeruginosa*, and 1 *Citrobacter* spp). These results were classified as true negatives and are included in the Figure in the Concordant T2R results block.

**Table 1.** Blood Cultures Positive With Antimicrobial Susceptibility Testing but Negative With T2Resistance Panel Testing Compared With Next-Generation Sequencing of Bacterial Isolates

Patient No., n=8	Positive Blood Culture	Antimicrobial Susceptibility Testing MIC, μg/mL	T2R Testing Result	Gene Detected With NGS	T2R Testing Interpretation
356	*Escherichia coli*	Ampicillin-sulbactam=16 I	None detected	None detected	True negative
482	MRSE	Oxacillin >2 R	None detected	*mec*A/C	False negative
522-1	MRSE	Oxacillin >2 R	None detected	*mec*A/C	False negative
522-2	*Staphylococcus capitis*	Oxacillin >2 R	None detected	*mec*A/C	False negative
583	*Escherichia coli*	Amoxicillin-clavulanate >16/8 R, ampicillin-sulbactam >16/8 R, cefazolin >16 R	None detected	None detected	True negative
681	MRSE	Oxacillin >2 R	None detected	*mec*A/C	False negative
755	*Pseudomonas aeruginosa*	Cefepime >16 R	None detected	None detected	True negative
756	MRSE	Oxacillin >2 R	None detected	*mec*A/C	False negative
886	*Citrobacter* spp	Amoxicillin-clavulanate=16/8 R, ampicillin-sulbactam=16/8 R, cefazolin >16 R	None detected	None detected	True negative

Note: Patient 522 had 2 isolates: methicillin-resistant *Staphylococcus epidermidis* and *Staphylococcus capitis*.

I, intermediate; MIC, minimum inhibitory concentration; MRSE, methicillin-resistant *Staphylococcus epidermidis*; NGS, next-generation sequencing; No., number; R, resistant; T2R, T2Resistance Panel.

Compared to AST, for gram-negative bacilli, the 31 T2R testing results showed 100% sensitivity for 2 *bla*_CTX-M-14/15_ genes detected (*K pneumoniae* and *E coli*); the remaining gram-negative resistance genes (*bla*_KPC_, *bla*_NDM_, *bla*_VIM_, *bla*_IMP_, and *bla*_OXA-48_, AmpC *bla*_CMY/DHA_) were not identified in 14 gram-negative bacilli, so sensitivity could not accurately be determined. Specificity was 100% for all 16 gram-negative bacilli. Enterococci (n=3) showed 100% sensitivity (1 *van*A/B gene detected *E faecium*) and 100% specificity. *S aureus* (n=2) showed 100% sensitivity (1 *mec*A/C gene detected methicillin-resistant *S aureus* and 1 *mec*A/C gene was not detected for methicillin-susceptible *S aureus*) and 100% specificity. However, coagulase-negative staphylococci (n=10) showed 17% sensitivity and 100% specificity (Figure).

An additional 100 sequential patients having an original negative blood culture as well as all negative cultures (blood and non-blood cultures collected within ±48 hours of the simultaneous blood culture/T2R sample) were selected from the 489 negative blood cultures. The original blood culture/T2R sample was tested with the T2R Panel. No antibiotic resistance genes were detected, and no false positives were detected (Figure, Negative BC tested with T2R). The overall specificity, including all 13 antibiotic resistance genes on the T2R Panel, was 100%.

### Analysis of Additional Positive Blood Cultures Collected Within ±48 Hours of the Negative Simultaneous Blood Cultures

Twenty-seven patients had a negative simultaneous blood culture (collected at the same day/time as the T2R sample) but then had an additional different positive blood culture within ±48 hours of the original simultaneous blood culture. The original simultaneous blood culture/T2R samples were tested to determine whether the T2R Panel could detect antibiotic resistance genes still circulating in the patient's bloodstream within 48 hours of the original negative blood culture. Three additional resistance genes were detected from 3 patients’ samples: 2 *van*A/B genes from 2 patients who had positive blood cultures that grew vancomycin-resistant *E faecium* and were collected the same day (but different times) as the original T2R samples; and 1 *bla*_CTX-M-14/15_ gene from a patient with a positive blood culture that grew *K pneumoniae* (resistant to ceftriaxone, cefazolin, and cefepime) and was collected 24 hours before the original T2R sample (Table 2).

**Table 2.** Analysis of Additional Positive Blood Cultures Collected Within ±48 Hours of the Negative Simultaneous (With the T2R Sample) Blood Cultures

Patient No., n=27	Simultaneous Blood Culture Collected With T2R Sample	Positive Blood Culture Collected Within ±48 Hours of Simultaneous Blood Culture	Time Positive Blood Culture Collected Compared to Simultaneous Blood Culture	T2R Testing Result	Antimicrobial Susceptibility Testing MIC Interpretation
49	No growth	MSSA	–48 h	None detected	Oxacillin S
79	No growth	*Pseudomonas aeruginosa*	–48 h	None detected	All drugs S
199	No growth	MSSA	Same day	None detected	Oxacillin S
218	No growth	*Escherichia coli*	–24 h	None detected	All drugs S
229	No growth	*Enterococcus faecalis*	–24 h	None detected	Vancomycin S
231	No growth	MSSE	–48 h	None detected	Oxacillin S
322	No growth	*Enterococcus faecium*	Same day	***van*A/B**	**Vancomycin R**
340	No growth	*Escherichia coli*	–48 h	None detected	All drugs S
352	No growth	*Enterococcus faecium*	–48 h and same day	***van*A/B**	**Vancomycin R**
358	No growth	*Escherichia coli*	–48 h	None detected	Ampicillin-sulbactam R, cefazolin I
363	No growth	MSSA	–48 h	None detected	Oxacillin S
426	No growth	*Acinetobacter baumannii*	+48 h	None detected	All drugs S
474	No growth	MSSA	–24 h, –48 h	None detected	Oxacillin S
545	No growth	*Escherichia coli, Vibrio parahaemolyticus*	–48 h	None detected	*E coli*–all drugs S; *V parahaemolyticus*–cefazolin I
553	No growth	MRSA	–24 h	None detected	Oxacillin R
636	No growth	MSSA	–48 h	None detected	Oxacillin S
678	No growth	*Enterococcus faecalis*	–24 h	None detected	Vancomycin S
690	No growth	*Klebsiella pneumoniae*	–24 h	None detected	All drugs S
726	No growth	*Serratia marcescens*	–48 h	None detected	Ceftriaxone, piperacillin-tazobactam I
744	No growth	*Klebsiella oxytoca*	–24 h	None detected	Cefazolin R
766	No growth	*Proteus mirabilis*	–48 h	None detected	All drugs S
794	No growth	MSSA	Same day	None detected	Oxacillin S
798	No growth	*Klebsiella pneumoniae*	–24 h	***bla*_CTX-M-14/15_**	**Ceftriaxone, cefazolin, cefepime R**
805	No growth	*Klebsiella pneumoniae*	–24 h, –48 h	None detected	Ampicillin-sulbactam, cefazolin R
828	No growth	*Enterococcus faecalis*	–24 h	None detected	Vancomycin S
856	No growth	MRSA	–24 h	None detected	Oxacillin R
857	No growth	MSSA	–48 h	None detected	Oxacillin S

Note: The T2Resistance Panel was tested using the simultaneous blood culture/T2R sample and compared to antimicrobial susceptibility testing for the nonsimultaneous blood culture (collected within ±48 hours of the simultaneous blood culture). Resistance genes detected are identified with bold.

h, hours; I, intermediate; MIC, minimum inhibitory concentration; MRSA, methicillin-resistant *Staphylococcus aureus*; MSSA, methicillin-susceptible *Staphylococcus aureus*; MSSE, methicillin-susceptible *Staphylococcus epidermidis*; No., number; R, resistant; S, susceptible; T2R, T2Resistance Panel.

### Time to Results for Antimicrobial Susceptibility Testing vs Results of T2R Testing

We compared the time to results for AST and T2R testing using a start time of instrument loading (Table 3). To obtain a direct comparison of instrument processing time, we did not include the time between sample collection and instrument loading. The mean ± SD time to AST results was 70.8 ± 16.6 hours, with a range of 46.1 to 117.2 hours. In contrast, the mean ± SD time to T2R results was 6.7 ± 1.5 hours, with a range of 3.6 to 9.8 hours. The mean difference in time between a T2R result and the AST result was 64.1 hours.

**Table 3.** Time to Results for Antimicrobial Susceptibility Testing vs the T2Resistance Panel

Test Method	Time to Results, hours, mean ± SD	Time to Results Range, hours
Antimicrobial susceptibility testing (MIC)	70.8 ± 16.6	46.1-117.2
T2Resistance Panel	6.7 ± 1.5	3.6-9.8
Difference	64.1	42.5-107.4

MIC, minimum inhibitory concentration.

### Potential Impact of T2R Testing on Patient Care

For each positive simultaneous blood culture sample tested with the T2R Panel (n=27), we assessed the timing of the T2R result relative to the reported AST and administered antibiotics and classified each patient into one of 4 impact-level categories (Table 4). The *no impact* (*none*) group included 11 patients for whom the T2R results would have had little to no impact; these patients were on appropriate therapy. The *some impact* group included 11 patients treated with an effective antimicrobial at the time of the T2R result, but the number of administered antibiotics could have been reduced. The *moderate impact* group included 3 patients who were administered therapy at the time of the T2R result but could have been switched to specific directed therapy earlier based on the results of T2R testing, which could have reduced the time to effective therapy. The *negative impact* group included 4 patients with coagulase-negative *Staphylococcus* treated with vancomycin that may have been discontinued or not administered if T2R testing had detected the *mec*A/C gene. Two patients (numbers 482 and 691) were each considered to have 2 potential impacts.

**Table 4.** Potential Impact of T2Resistance Panel Testing for Patients with a Positive Blood Culture and Antimicrobial Susceptibility Testing Results

Patient No., n=27	Date and Time Blood Culture/T2R Sample Collected	T2R Testing Result; Date and Time Available	Positive Blood Culture–AST Result; Date and Time Available	Potential Impact of T2R Testing Result on Antibiotic Therapy	Impact Level of T2R Testing Result^a^
1	1/13/21 at 9:17	None detected; 1/13/21 at 13:12	*Escherichia coli*–all drugs S; 1/15/21 at 13:02	Vancomycin would not have been administered (1/14/21-1/17/21).	Some
82	2/9/21 at 12:50	None detected; 2/9/21 at 19:55	*Raoultella ornithinolytica*–all drugs S; 2/12/21 at 10:55	On appropriate therapy (cefepime 2/9/21-2/11/21; tobramycin 2/10/21).	None
109	2/19/21 at 9:02	*mec*A/C; 2/19/21 at 12:59	*Staphylococcus aureus*–oxacillin R; 2/21/21 at 9:33	On appropriate therapy (vancomycin 2/19/21-2/21/21; cefazolin 2/19/21-2/20/21).	None
119	2/25/21 at 11:00	None detected; 2/25/21 at 14:29	*Enterococcus faecium*–vancomycin S; 2/27/21 at 11:37	On appropriate therapy, (vancomycin 2/25/21-2/27/21) but piperacillin-tazobactam (2/25/21-2/27/21) may have been discontinued.	None
153	3/2/21 at 12:12	None detected; 3/2/21 at 18:15	*Staphylococcus cohnii*–oxacillin S; 3/2/21 at 18:15	Vancomycin would not have been administered (3/9/21-3/15/21).	Some
288	4/6/21 at 11:35	None detected; 4/6/21 at 17:49	*Escherichia coli*–all drugs S; 4/8/21 at 11:03	Vancomycin would not have been administered (4/7/21-4/8/21).	Some
318	4/8/21 at 12:14	None detected; 4/8/21 at 19:00	*Plesiomonas shigelloides*–all drugs S; 4/10/21 at 11:44	On appropriate therapy (cefepime 4/8/21-4/9/21), but patient died 4/9/21.	None
356	4/19/21 at 11:12	None detected; 4/19/21 at 20:45	*Escherichia coli*–all drugs S; 4/21/21 at 10:35	On appropriate therapy (ciprofloxacin 4/21/21-5/4/21).	None
395	5/14/21 at 9:01	*mec*A/C; 5/14/21 at 12:48	*Staphylococcus epidermidis*–oxacillin R; 5/17/21 at 9:59	96 hours faster time to effective therapy (vancomycin).	Moderate
427	5/24/21 at 19:53	None detected; 5/24/21 at 19:53	*Staphylococcus cohnii*–oxacillin S; 5/27/21 at 14:02	Vancomycin would not have been administered (5/25/21-5/26/21).	Some
482	6/18/21 at 9:15	*van*A/B; 6/18/21 at 16:25	*Enterococcus faecium*–vancomycin R; *Staphylococcus epidermidis*–oxacillin R; 6/22/21 at 8:26	79.2 hours faster time to effective therapy (daptomycin). Vancomycin may have been discontinued (6/19/21-6/20/21).	Moderate Negative
483	6/17/21 at 9:41	None detected; 6/17/21 at 15:33	*Escherichia coli*–all drugs S; 6/19/21 at 7:48	On appropriate therapy (ceftriaxone 6/18/21-6/19/21; ciprofloxacin 6/20/21-7/1/21).	None
512	6/28/21 at 14:44	None detected; 6/28/21 at 20:59	*Staphylococcus epidermidis*–oxacillin S; 7/2/21 at 10:42	Vancomycin would not have been administered (6/29/21-7/4/21).	Some
521	6/24/21 at 10:38	None detected; 6/24/21 at 14:13	*Klebsiella pneumoniae*–all drugs S; 6/27/21 at 10:28	Vancomycin may not have been administered (6/25/21).	Some
522	6/30/21 at 18:06	None detected; 6/30/21 at 23:20	*Staphylococcus epidermidis*–oxacillin R; *Staphylococcus capitis*–oxacillin R; *Staphylococcus haemolyticus*–oxacillin S; 7/5/21 at 12:04	Vancomycin may not have been administered (7/1/21-7/2/21).	Negative
583	7/28/2021 at 11:00	None detected; 7/28/21 at 16:08	*Escherichia coli*–amoxicillin-clavulanate, ampicillin-sulbactam, cefazolin R; 8/1/21 at 9:17	Vancomycin may not have been administered (7/28/21-7/29/21).	Some
615	8/2/21 at 14:32	None detected; 8/2/21 at 21:40	*Staphylococcus aureus*–oxacillin S; 8/4/21 at 11:17	Vancomycin would not have been administered (8/4/21-8/7/21).	Some
647	8/2/21 at 18:13	None detected; 8/3/21 at 2:40	*Enterococcus faecalis*–vancomycin S; 8/5/21 at 14:15	On appropriate therapy (doxycycline 8/2/21-8/12/21).	None
661	8/10/21 at 13:46	None detected; 8/10/21 at 22:31	*Enterobacter cloacae*–all drugs S; 8/14/21 at 9:10	Vancomycin would not have been administered (8/11/2021).	Some
681	8/11/21 at 15:20	None detected; 8/11/21 at 1:07	*Staphylococcus epidermidis*–oxacillin R; 8/14/21 at 11:18	Vancomycin may not have been administered (8/12/21-8/16/21; 8/18/21).	Negative
691	8/12/21 at 11:01	*bla*_CTX-M-14/15_; 8/12/21 at 18:24	*Klebsiella pneumoniae*–ampicillin-sulbactam I; cefazolin and ceftriaxone R; 8/14/2021 at 10:03	Vancomycin would not have been administered (8/17/21; 8/19/21-8/20/2021). 120 hours faster time to effective therapy (meropenem).	Some Moderate
724	8/18/21 at 17:07	None detected; 8/18/21 at 2:34	*Proteus mirabilis*–all drugs S; 8/21/21 at 10:56	Cefepime would not have been administered (8/19/21-8/23/21).	Some
755	8/23/21 at 14:16	None detected; 8/23/21 at 22:42	*Pseudomonas aeruginosa*–cefepime R; 8/26/21 at 11:25	Patient died the same day as treated with cefepime and vancomycin (8/23/21).	None
756	8/23/21 at 11:19	None detected; 8/23/21 at 21:06	*Staphylococcus epidermidis*–oxacillin R; 8/26/21 at 10:07	Vancomycin may not have been administered (8/24/21-8/31/21).	Negative
776	8/26/21 at 18:17	None detected; 8/26/21 at 1:46	*Klebsiella pneumoniae*–ampicillin-sulbactam R; 8/31/21 at 9:00	On appropriate therapy (piperacillin-tazobactam 8/26/21-8/31/21).	None
836	9/16/21 at 12:59	None detected; 9/16/21 at 20:35	*Escherichia coli*–all drugs S; 9/19/21 at 20:35	On appropriate therapy (ceftriaxone 9/17/21-10/5/21; amoxicillin-clavulanate 9/19/21).	None
886	10/4/21 at 10:45	None detected; 10/4/21 at 18:06	*Citrobacter* spp–amoxicillin-clavulanate, ampicillin-sulbactam, and cefazolin R; 10/7/21 at 10:36	On appropriate therapy (piperacillin-tazobactam 10/4/21-10/6/21).	None

^a^Explanation of impact levels: *None*, patients were on appropriate therapy; *Some*, patients were treated with an effective antimicrobial at the time of the T2R testing result, but the number of administered antibiotics could have been reduced; *Moderate*, patients were being administered therapy at the time of the T2R testing result but could have been switched to specific directed therapy earlier based on the results of the T2R Panel, which could have reduced the time to effective therapy; *Negative,* patients with methicillin-resistant coagulase-negative *Staphylococcus* were being administered vancomycin that may have been discontinued or not administered if T2R testing had not detected a *mec*A/C gene.

AST, antimicrobial susceptibility testing; I, intermediate; No., number; R, resistant; S, susceptible; T2R, T2Resistance Panel.

## DISCUSSION

In this investigator-initiated study, we demonstrated that T2R testing could rapidly and accurately identify the antibiotic resistance genes on the T2R Panel in patients’ blood samples when compared to the AST performed for a positive blood culture. In 13/27 (48%) of patients tested, the T2R Panel showed the ability to favorably impact antibiotic therapy. Comparable results were found in 2 studies in Italy that evaluated the T2R Panel.^18,19^ This culture-independent diagnostic panel could potentially inform the clinician of the presence or absence of the 13 antibiotic resistance genes in as little as 3.6 to 9.8 hours after the samples were loaded into the instrument compared to the AST range of 46.1 to 117.2 hours, thereby reducing the time to effective therapy.

However, improvement is needed for *mec*A/C detection in coagulase-negative staphylococci. The low sensitivity (17%) for detection of the *mec*A/C genes in coagulase-negative staphylococci seen in this study is concerning and should be addressed by T2 Biosystems.

This T2R Panel and the T2Bacteria Panel are designed to be ordered with a blood culture to supplement the standard of care. Ideally, a blood sample for the T2R Panel and the T2Bacteria Panel would be collected at the same time as the blood culture sample. The T2R Panel and T2Bacteria Panel would be tested immediately, with results available in 3 to 5 hours, whereas the blood culture bottles would have to be incubated in an instrument until growth is detected (usually for at least 24 hours) before reporting as a positive. Other rapid tests that provide identification and the presence of resistance genes are available but require testing the blood culture broth after it has been detected as positive.

Because blood culture results—identification and susceptibility results for the organism—may not typically be reported for 3 to 4 days, empiric therapy to cover a broad range of potential bacterial pathogens is administered to septic patients. Any aid to direct early appropriate targeted therapy and reduce morbidity or mortality would be beneficial.^3,4^ The prolonged use of inappropriate antibiotics often leads to concerns of toxicity for the patient, development of antibiotic resistance by the organism, and increased costs for the patient (pharmacy and hospital length of stay).^1,2^

### Limitations

This T2R Panel clinical study was noninterventional, so the authors were unable to assess the effect of the test on patient outcomes. In addition, a total of 547 patients were enrolled, but only 58 blood cultures were positive. Of these 58 positive blood cultures, 18 were probable contaminants and excluded from analysis. A total of 27 positive blood cultures with T2R Panel results were evaluated. Within this group, the T2R Panel was positive for 5 resistance genes: 2 *bla*_CTX-M-14/15_ (*K pneumoniae* and *E coli*), 2 *mec*A/C (1 methicillin-resistant *S aureus,* 1 methicillin-resistant *S epidermidis*), and 1 *van*A/B (*E faecium*). Because of the small number of resistant gram-negative organisms in this study, *bla*_KPC_, *bla*_NDM_, *bla*_VIM_, *bla*_IMP_, and *bla*_OXA-48_, and AmpC *bla*_CMY_ and *bla*_DHA_ were not detected, limiting our ability to accurately report the clinical sensitivity of the T2R Panel, especially for the gram-negative resistance genes not detected in any of the samples.

A larger study is needed to include differences in patient populations and patient care. A multicenter, prospective clinical trial (NCT05231187) with a newly formulated T2R Panel was conducted in the United States from January 2022 to November 2023 and will provide differences in patient populations and site-to-site differences in patient care. The T2R Panel identifies most, but not all, common genetic resistance markers. Therefore, a blood culture including susceptibility testing of a bacterial isolate is still necessary.

## CONCLUSION

The T2R Panel can detect certain antibiotic resistance genes from gram-positive and gram-negative bacterial bloodstream infections in hours vs the days required for a positive blood culture with susceptibility testing. The use of the T2R Panel in combination with the T2Bacteria Panel has the potential to improve current diagnostic testing by influencing initial treatment decisions, including a possibly faster transition to targeted therapy and faster de-escalation of empiric therapy. Other potential benefits could be shorter hospital stays for patients and help with efforts in antimicrobial stewardship. Additional studies with larger numbers of samples are warranted.

## Notes

*This article meets the Accreditation Council for Graduate Medical Education and the American Board of Medical Specialties Maintenance of Certification competencies for Patient Care, Medical Knowledge, Systems-Based Practice, and Practice-Based Learning and Improvement.*
