# ERRATUM

**DOI:** 10.31486/toj.25.5054

**Published:** 2025

Ashcraft D, Pierre JS, Davis H, Scariano S, Latsis M, Vortisch R, Smith S, Pankey G. Evaluation of the diagnostic accuracy of the T2Resistance Panel (research use only) in patients with possible bacterial bloodstream infections. *Ochsner J*. 2025 Spring;25(1):24-33. doi: 10.31486/toj.24.0101. PMID: 40123928; PMCID: PMC11924974.

Erratum submitted by the authors. The authors wish to correct three errors in the referenced article.

First, the (c) entry in the following sentence is incorrect:

The T2Resistance (T2R) Panel (T2 Biosystems, Inc) (available for research use only in the United States, pending clearance by the FDA for use as a diagnostic test) uses the same technology for direct detection of 13 antibiotic resistance genes from any bacterial species, with sensitivity as low as ≤10 CFU/mL: (a) the major carbapenem resistance genes listed in the “2019 Antibiotic Resistance Threats Report” from the Centers for Disease Control and Prevention^16^ (*bla*_KPC_, *bla*_NDM_, *bla*_VIM_, *bla*_IMP_, and *bla*_OXA-48_); (b) the extended-spectrum beta-lactamases (*bla*_CTX-M-14_ and *bla*_CTX-M-15_); (c) the beta-lactamases active against ampicillin carbapenemase (AmpC *bla*_CMY_ and *bla*_DHA_); (d) the gram-positive *van*A and *van*B resistance genes that are responsible for vancomycin resistance in *Enterococcus*; and (e) the *mec*A and *mec*C resistance genes that are responsible for methicillin resistance in *Staphylococcus*. T2R testing results are available in 3 to 5 hours.^17^

Item (c) in the above sentence should read as follows: (c) other beta-lactamases active against some beta-lactams (AmpC *bla*_CMY_ and *bla*_DHA_).

Second, the following sentence contains an error:

The *negative impact* group included 4 patients with coagulase-negative *Staphylococcus* treated with vancomycin that may have been discontinued or not administered if T2R testing had detected the *mec*A/C gene.

The above sentence should read as follows:

The *negative impact* group included 4 patients with coagulase-negative *Staphylococcus* treated with vancomycin that may have been discontinued or not administered if T2R testing had reported the *mec*A/C gene as not detected.

Third, one of the authors' names is shown incorrectly in the PubMed citation. The name Pierre JS should instead read St Pierre J.

